# Supplementary figures and images for: Hydralazine Sensitizes to the Antifibrotic Effect of 5-Aza-2′-deoxycytidine in Hepatic Stellate Cells
Source: Biology (Basel). 2020 Jun 3;9(6):117. doi: 10.3390/biology9060117 (PMC7345531; doi:10.3390/biology9060117)

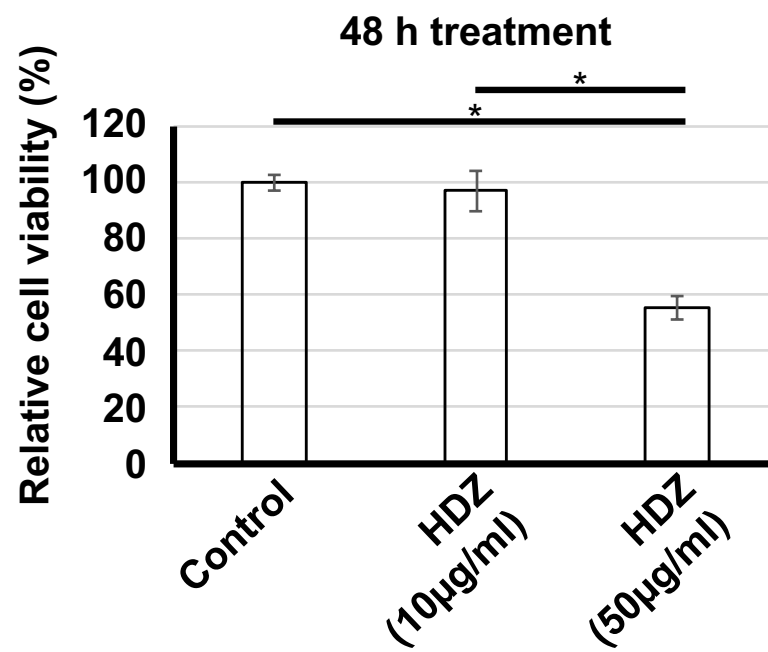

**Supplementary Figure 1**

Supplement: Supplementary file 1 [file biology-09-00117-s001.pdf]
